# Absorption of the Iris

**Published:** 1831

**Authors:** 


ABSORPTION OF THE IRIS.

A BOY received a blow upon the eye with a piece of metal, lacerating the iris, and followed by considerable pain and inflammation. For a time the pupil was cordiform, being pointed at its lower part, but slowly, and without pain, the whole iris was absorbed, and the eye became amaurotic. Mr. Middlemore has several times observed laceration of the iris followed by partial, in one instance by its total absorption. In every case the injured eye became amaurotic.—*Mid. Report.* No. XI.

---

XIII.

